# The diagnostic performance of ^18^F-FAMT PET and ^18^F-FDG PET for malignancy detection: a meta-analysis

**DOI:** 10.1186/s12880-017-0237-1

**Published:** 2017-12-28

**Authors:** Arifudin Achmad, Anu Bhattarai, Ryan Yudistiro, Yusri Dwi Heryanto, Tetsuya Higuchi, Yoshito Tsushima

**Affiliations:** 1 Department of Diagnostic Radiology and Nuclear Medicine, Gunma University Graduate School of Medicine, 3-39-22 Showa-machi, Maebashi, Gunma 371-8511 Japan; 2 Department of Nuclear Medicine and Molecular Imaging, Faculty of Medicine, Padjadjaran University, Jl. Professor Eyckman No.38, Bandung, West Java 40161 Indonesia; 3 Department of Nuclear Medicine, Mochtar Riady Comprehensive Cancer Center, Jl. Garnisun Dalam No. 2–3, Semanggi, Jakarta, 12930 Indonesia

**Keywords:** ^18^F-FAMT, ^18^F-FDG, Malignancy, Meta-analysis, Diagnostic accuracy

## Abstract

**Background:**

This meta-analysis aims to compare the diagnostic performance of l-3-^18^F-α-methyl tyrosine (^18^F-FAMT) positron emission tomography (PET) and 2-deoxy-2-[^18^F]fluoro-d-glucose (^18^F-FDG) PET for malignancy detection.

**Methods:**

The workflow of this study follows Cochrane Collaboration Guidelines of a systematic review of diagnostic test accuracy studies. An electronic search was performed for clinical diagnostic studies directly comparing ^18^F-FAMT and ^18^F-FDG PET for malignant tumors. Study quality, the risks of bias and sources of variation among studies were assessed using the QUADAS (Quality Assessment of Diagnostic Accuracy Studies) assessment tool. A separate meta-analysis was performed for diagnostic performance based on visual assessment and diagnostic cut-off values. Whenever possible, a bivariate random-effect model was used for analysis and pooling of diagnostic measures across studies.

**Results:**

Electronic search revealed 56 peer-reviewed basic science investigations and clinical studies. Six eligible studies (272 patients) of various type of cancer were meta-analyzed. The ^18^F-FAMT diagnostic accuracy for malignancy was higher than ^18^F-FDG based on both visual assessment (diagnostic odd ratio (DOR): 8.90, 95% confidence interval (CI) [2.4, 32.5]) vs 4.63, 95% CI [1.8, 12.2], area under curve (AUC): 77.4% vs 72.8%) and diagnostic cut-off (DOR: 13.83, 95% CI [6.3, 30.6] vs 7.85, 95% CI [3.7, 16.8], AUC: 85.6% vs 80.2%), respectively. While the average sensitivity and specificity of ^18^F-FAMT and ^18^F-FDG based on visual assessment were similar, ^18^F-FAMT was significantly more specific than ^18^F-FDG (*p* < 0.05) based on diagnostic cut-off values.

**Conclusions:**

^18^F-FAMT is more specific for malignancy than ^18^F-FDG, while their sensitivity is comparable. ^18^F-FAMT PET is equal to ^18^F-FDG PET in diagnostic performance for malignancy detection in several cancer types.

## Background

Since its introduction as a positron emission tomography (PET) tracer back in the early 1970’s, [^18^F]-fluorodeoxyglucose (^18^F-FDG) has been widely utilized and now comprises more than 96% of PET studies worldwide [1]. Even though ^18^F-FDG is mainly a radiotracer for oncology, it is not a tumor-specific PET tracer, since it is essentially based on the presence of elevated glucose uptake [2]. Many malignant lesions, in fact, are poorly imaged with ^18^F-FDG; some due to their slow growth or low metabolic nature, and others due to their location within highly metabolic organs such as the brain and liver [3]. Various alternative PET tracers have been synthesized and evaluated over the last decade to overcome the limitations of ^18^F-FDG, including tracers based on amino acid metabolism such as l-3-^18^F-α-methyl tyrosine (^18^F-FAMT) [1, 4].

^18^F-FAMT has been validated in several clinical studies to be useful for the prediction of cancer prognosis and to rule out benign lesions from malignant neoplasms [5–13]. The tumor accumulation of ^18^F-FAMT is exclusively facilitated by the L-type amino acid transporter 1 (LAT1), which is highly upregulated in malignant cells [14]. Unlike other amino acid PET tracers that are not specific to a single amino acid transporter, ^18^F-FAMT has a α-methyl moiety that allows it to be transported only by LAT1, making it highly specific for malignancies [15]. Although a handful of clinical studies have investigated its potential in malignant tumor detection, the overall diagnostic performance of ^18^F-FAMT remains unknown. The present meta-analysis aimed to determine the diagnostic performance of ^18^F-FAMT PET for detection and evaluation of malignant lesions in a direct side-by-side comparison to ^18^F-FDG PET.

## Methods

### Search strategy and study selection

The design of this study followed the current recommendations for systematic review of diagnostic test accuracy studies from the Cochrane Collaboration [16, 17]. Studies evaluating ^18^F-FAMT PET or PET/CT as a diagnostic tool for evaluation of malignancy were electronically searched in Pubmed/MEDLINE, Web of Science, ScienceDirect, and Google Scholar databases from the inception of ^18^F-FAMT to December 2016 without language restriction. The search algorithm was based on a combination of the following terms: ^18^F-FAMT or ^18^F-FMT or “alpha-methyltyrosine.” To find more potential studies, we also screened references of the retrieved studies. Articles without raw clinical data such as reviews, conference abstracts, editorial, comments, preclinical, animal and non-radiopharmaceutical studies, or clinical studies with fewer than ten patients were excluded. The following information was extracted: first author’s name, year of publication, study design, study population, types/subtypes of malignancies, injected dose, imaging parameters, cut-off values of quantitative parameters, study and follow-up period, final diagnosis, and the reference standard.

The clinical studies obtained were subject to inclusion criteria for further analysis: (a) both ^18^F-FAMT and ^18^F-FDG were used to differentiate malignant tumors from benign lesions, (b) histopathological analysis and/or close clinical and imaging follow-up were used as reference standards, (c) when data or subsets of data were presented in more than one article, the article with the most detailed/recent data was chosen, and (d) only articles in which at least 10 of the 14 questions in the QUADAS (Quality Assessment Tool for Diagnostic Accuracy Studies) questionnaire were answered ‘yes’ were included [18]. Studies were screened for eligibility, the risk of bias, and source of variations by three authors (AA, AB, RY) independently. Disagreements regarding the eligibility of a study were resolved by consensus.

### Meta-analysis

Meta-analysis of the diagnostic performance of ^18^F-FAMT and ^18^F-FDG in recognizing malignancies was performed following the current recommendations [17] and was conducted separately for two diagnostic methods: 1) by visual assessment, and 2) by diagnostic cut-off values applied in each study. From each study included, the number of true positives, false positives, true negatives, and false negatives were extracted to construct a 2 × 2 contingency table. If studies lacked clear data to produce such tables, the first authors were contacted when possible. This main data were described on forest plots of specificity and sensitivity.

Heterogeneity and between-study variability were evaluated, and subgroup study (meta-regression analysis) was used to investigate the source, if any. A Higgins’ inconsistency *I* ^*2*^ up to 30% was considered little evidence of heterogeneity. To determine whether different thresholds were used to define positive and negative test results (either explicitly or implicitly), the Spearman *ρ* between the logit of sensitivity and logit of 1 − specificity was calculated to assess the presence of a threshold effect. A strong positive correlation (Spearman *ρ* > 0.6) would suggest the presence of a threshold effect. Whenever possible, a bivariate random-effect model meta-analysis method was used to obtain summary estimates of sensitivity and specificity across studies instead of univariate approaches.

The hierarchical summary of the receiver operating characteristic (HSROC) curve was plotted following the method of Rücker and Schumacher [19]. The area under the curve (AUC), which is the average true-positive rate over the entire range of false-positive rate, serves as a global measure of test performance, while the diagnostic odd ratio (DOR) is calculated to describe the diagnostic value [20]. Note that the DOR is a single overall indicator of diagnostic performance and is, unlike sensitivity and specificity, independent of any threshold value. Meta-analysis was performed using the ‘mada’ (Meta-Analysis of Diagnostic Accuracy) package in R statistical software version 3.2.2 [21, 22].

## Results

### Literature search

**Fig. 1:**
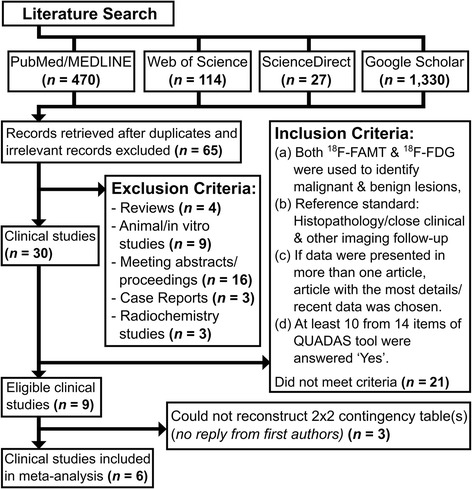
The study selection

The systematic search was performed to collect diagnostic test studies using ^18^F-FAMT and ^18^F-FDG PET for malignancy detection. The search yielded 65 studies involving ^18^F-FAMT as PET radiotracer in basic science investigations and clinical studies. There were three radiochemistry studies, nine in vitro and animal studies, four review articles, and 49 clinical studies. Thirty studies among these 49 clinical studies were original articles in which both PET radiotracers were employed. Figure 1 summarizes the systematic study selection.

### Study eligibility, quality, and risk of bias

Nine eligible studies according to the inclusion criteria (Table 1) were further evaluated with QUADAS tool. All were prospective studies of good quality (QUADAS Scores >10) involving at least 19 patients (patient number range: 19–74) and 21 lesions (lesion number range: 21–75). Overall, the nine eligible studies had a low risk of bias, except in blinding from the index test results (Additional file 1: Table S1). Blinding from the index test results was sometimes unavoidable in the clinical workflow, since histopathological diagnosis is established after the primary surgery or biopsy, while PET imaging is an early step in workups to establish the clinical diagnosis. In one study, the histopathology (biopsy) diagnosis was known before the PET study was performed [7]. However, this study was later excluded from the meta-analysis (Table 1). The other important potential source of bias was the use of other imaging studies (CT, MRI or bone scans) and close clinical monitoring as verification methods in one study [5]. However, in this study, only two patients (from 19 patients, total 57 lesions) had their lesions diagnosed without any histological examination: one had malignant melanoma in the foot (single lesion), and the other had diffuse malignant melanoma (lesions in the brain and spinal cord).

**Table 1:** Characteristics of Diagnostic Comparison Studies of ^18^F-FAMT and ^18^F-FDG

Study (year) [Ref]	N	Mean/Median Age (range)	Sex (M/F)	Tumour & other pathology examined	No. of lesions	^18^F-FAMT dose	^18^F-FDG dose	PET studies interval	Follow-up period	Gold Standard & verification	Study design	Study Period	QUADAS Score^b^
Inoue (1999) [6]	20	41 ± 21 (1–71)	8/12	Brain tumour	23	185 MBq	200 MBq	Within 1 wk.	> 4 mo.	H (16), Img & Cln (4)	Pro	ND	12 (6,11)
Watanabe (2000) [12]^a^	74	44 (12–83)	37/38	Musculoskeletal tumours: 24 bone, 48 soft tissue	22 mlg, 53 bgn	185–350 MBq	185–350 MBq	ND	> 1 y.	H	Pro	2/‘98–6/‘99	13 (11)
Inoue (2001) [5]^a^	19	58 (20–84)	13/6	Lung cancer (10), mlg myeloma (2), Chondrosarcoma (1), Prostate (1), mlg lymphoma (1), mlg of unknown origin (1), Schwannoma (1), Sarcoidosis (2)	57	200–370 MBq	200–370 MBq	Within 1 wk.	> 8 mo.	H (31), Img & Cln (26)	Pro	ND	10 (1,6,7,11)
Sato (2003) [13]	14	Gliomatosis: 45 (15–60) Non-neoplastic: 41.2 (23–76)	4/4 5/1	- Gliomatosis cerebri (8): Anaplastic (1), grade II astrocytoma (4) grade III astrocytoma (3) - Non-neoplastic diseases (6)	ND	185 MBq	ND	1–5 wk. (detailed)	ND	H (8) for gliomatosis Img & Cln for non-neoplasms	Pro	ND	12 (6,11)
Suzuki (2005) [11]^a^	57	58.1 (27–87)	29/28	Fatty tissue tumour	32 lipoma, 25 liposarcoma	185–350 MBq	185–350 MBq	ND	ND	H (57)	Pro	9/‘97–12/‘03	12 (11,4Un)
Kaira (2007) [7]	41	61 (45–82) 43 (20–73)	13/4 9/15	Lung cancer (17) Sarcoidosis (24)	9 AC, 6 SQC, 2 NSCLC, 16 LNM, 24 sarcoidosis	4–5 MBq/kg	5–6 MBq/kg	ND for lung cancer. Sarcoidosis: 1 wk. after diagnosis established.	> 2 y.	H (41)	Pro	Sarcoidosis: 9/‘98–8/‘05	12 (10,12)
Miyakubo (2007) [9] ^a^	43	ND (31–90) ND (32–81)	16/20 5/2	Maxillofacial tumours: mlg. (36), bgn. (7) and LNM (14)	34 SQC, 1 rhabdomyosarcoma, 1 mucoepidermoid carcinoma, 14 LNM, 7 bgn	5–6 MBq/kg	5–6 MBq/kg	2 wk.	> 6 mo.	H (43)	Pro	5/‘99–7/‘06	13 (11)
Kaira (2009) [24]^a^	43	67 (41–79)	33/10	Thoracic tumours: mlg. (37), bgn. (6)	19 AC, 9 SQC, 1 LCC, 2 atypical SQC, 3 Bronchoalveolar carci-noma, 1 carcinoid, 6 bgn	ND	5 MBq/kg	1–14 d. (mean: 4 d.)	ND	H (43)	Pro	5/‘07–3/‘08	12 (11,12)
Tian (2011) [23]^a^	36	ND (11–84)	22/14	Musculoskeletal tumour	13 mlg, 23 bgn	260 MBq	320 MBq	Maximum 2 wk.	ND	H, Img & Cln (36)	Pro	ND	13 (11)

*Abbreviations: MBq/kg* (MegaBecquerel/kg), *d.* Days, *wk.* Weeks, *mo.* Months, *y.* Year, *H* Histopathology, *Img* Imaging, *Cln* Clinical follow-up, *Pro* Prospective, *Retro* Retrospective, *mlg* malignant, *bgn* benign, *LNM* lymph node metastases, *AC* adenocarcinoma, *SQC* squamous cell carcinoma, *NSCLC* non-small cell lung cancer, *LCC* large cell carcinoma, *ND* not determined

^a^Studies included in the meta-analysis

^b^QUADAS Score was presented as ‘total score (item number which answered ‘No’ or ‘Unclear’ *(Un)*)’. QUADAS tool items were described in Tablze S1

Six studies were included in the final meta-analysis due to the availability of individual patient data to construct 2 × 2 contingency tables (Table 1 and Additional file 1: Table S2). All studies employed maximum standardized uptake value (SUV_max_) for quantitative interpretation of the PET images. Four explicitly described SUVmax cut-off value for discrimination between malignant and benign lesions. The SUVmax cut-offs of ^18^F-FAMT studies ranged from 1 to 1.45 while in ^18^F-FDG studies, they ranged from 0.81 to 4.72. Six studies with a total sample size of 272 patients (278 lesions) with malignancy from musculoskeletal [12, 23], fatty tumors [11], maxillofacial tumors [9], lung cancer [24], and several different tumors [5] were included.

### Descriptive statistics

**Fig. 2:**
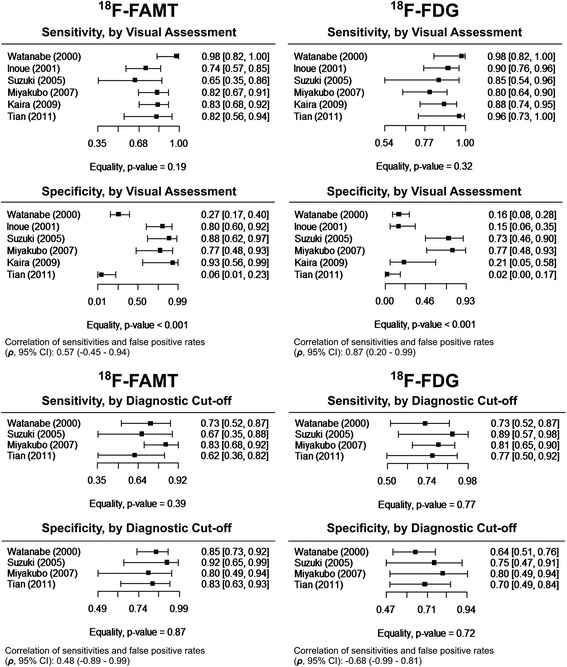
Sensitivity and specificity of ^18^F-FAMT and ^18^F-FDG for malignancy detection

Figure 2 described the paired sensitivity and specificity of ^18^F-FAMT and ^18^F-FDG of each study in forest plots. The sensitivity of both radiotracers was homogeneous either based on the visual assessment or diagnostic cut-off values. Their specificity was heterogeneous based on visual assessment. The Spearman correlation (*ρ*) between sensitivity and the logit of 1-specificity suggest that accuracy of both radiotracers based on visual assessment may be influenced by threshold effects (≥ 0.6). However, their accuracy was less affected by threshold effect when the diagnostic cut-off value was implemented.

### Meta-analysis

Due to the small number of studies included, both univariate and bivariate approach meta-analysis was performed. The bivariate approach is the method currently recommended; however, it cannot handle small sample sizes [17]. Meta-regression or subgroup analysis (to explore the source of heterogeneity) was also irrelevant due to the limited number of studies.

Table 2 described the summary estimates from the random effects univariate analysis. DOR of ^18^F-FAMT and ^18^F-FDG based on visual assessment were 8.90 and 4.63, while those based on diagnostic cut-off were 13.83 and 7.85, respectively. The heterogeneity between studies as well as inter-study was observed only mildly on ^18^F-FAMT studies based on visual assessment (Higgins’ *I* ^*2*^: 11.76%, *τ* ^*2*^: 1.46) while it was not observed in other studies.

**Table 2:** Summary estimates from univariate meta-analysis

Summary estimates (95% CI)	Based on visual assessment		Based on diagnostic cut-off	
	^18^F-FAMT	^18^F-FDG	^18^F-FAMT	^18^F-FDG
Between-study heterogeneity	I^2^: 11.76%	I^2^: 0%	I^2^: 0%	I^2^: 0%
Inter-study heterogeneity	τ^2^: 1.46	τ^2^: 0.30	τ^2^: 0.00	τ^2^: 0.00
DOR	8.90 (2.4–32.5)	4.63 (1.8–12.2)	13.83 (6.3–30.6)	7.85 (3.7–16.8)

The summary estimate measures of the random effects bivariate model are described in Table 3 **.** There was no significant difference in average sensitivity and specificity between ^18^F-FAMT and ^18^F-FDG based on visual assessment (*p* = 0.181 and 0.207, respectively). However, ^18^F-FAMT was significantly more specific than ^18^F-FDG (*p* < 0.01) based on diagnostic cut-off values. DOR of ^18^F-FAMT and ^18^F-FDG based on visual assessment were 8.33 and 3.88 while based on diagnostic cut-off were 16.70 and 8.17, respectively.

**Table 3:** Summary estimates from bivariate meta-analysis

Summary estimates (95% CI)	Based on visual assessment		Based on diagnostic cut-off	
	^18^F-FAMT	^18^F-FDG	^18^F-FAMT	^18^F-FDG
Average Sensitivity	80.7% (72.4–87.0%)	88.8% (80.2–93.9%)	74.1% (63.0–82.7%)	78.3% (67.8–86.1%)
*p* values	0.181		0.542	
Average Specificity	60.7% (25.3–87.6%)	29.2% (9.2–62.5%)	84.4% (75.7–90.4%)	68.1% (58.1–76.6%)
*p* values	0.207		0.009	
Positive Likelihood	2.46 (1.11–6.23)	1.34 (1.00–2.25)	4.90 (2.96–7.92)	2.48 (1.81–3.44)
Negative Likelihood	0.36 (0.20–0.70)	0.44 (0.20–0.98)	0.31 (0.20–0.45)	0.33 (0.20–0.49)
DOR	8.33 (1.60–26.10)	3.88 (1.02–10.40)	16.70 (7.25–33.40)	8.19 (3.86–15.40)
AUC^a^	77.4%	72.8%	85.6%	80.2%
λ (mean accuracy)	3.81	3.08	3.44	2.99

^a^approximated following Rucker-Schumacher’s method [19]

**Fig. 3:**
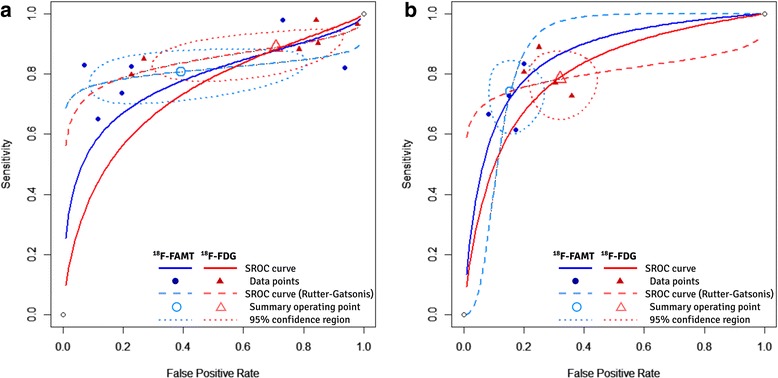
Summary ROC plots obtained from the bivariate model of the diagnostic performance of ^18^F-FAMT and ^18^F-FDG based on (**a**) visual assessment and (**b**) diagnostic cut-off value. Oval regions are the 95% confidence regions around the summary operating points. The SROC curves from parametrization according to Rutter and Gatsonis are also presented

The HSROC curves of diagnostic performance comparison are shown in Fig. 3. The AUC of diagnostic performance of ^18^F-FAMT and ^18^F-FDG based on visual assessment was 77.4% and 72.8%, while those based on diagnostic cut-off were 85.6% and 80.2%, respectively. The estimated SROC curves from the bivariate model (Rutter-Gatsonis method) were also plotted as a reference (Fig. 3, *dashed lines*). The summary operating points of ^18^F-FAMT were on the left side of those of ^18^F-FDG in both HSROC curves comparison, which indicated that ^18^F-FAMT provided more specificity. Meanwhile, their similar heights of the summary operating points on the Y-axis showed that their sensitivities were comparable.

## Discussion

This meta-analysis summarized the diagnostic performance of ^18^F-FAMT PET for detection of various malignancies in six studies with total 278 patients. Overall, the included studies have a low risk of bias with good methodological quality based on QUADAS tool. Our results demonstrated that ^18^F-FAMT is comparable with ^18^F-FDG for its diagnostic performance in detecting malignancies by either visual assessments or diagnostic cut-off values. Moreover, ^18^F-FAMT capability is coherent in several types of tumors, where all individual diagnostic test studies directly compared the two radiotracers on the same patients in a prospective study design. Additionally, the potential for selection bias can be safely ignored due to the sufficient number of lesions evaluated in each study included (*n* > 20). Another strength of this meta-analysis is that even though the study number is limited, heterogeneity was not substantial. The source of observed mild heterogeneity was likely due to threshold effects, which was found in studies based on visual assessment. However, other potential sources of heterogeneity should not be neglected since subgroup analysis was not applicable [25]. Publication bias is an important consideration in any meta-analysis. However, DOR heterogeneity observed in our results precludes the necessity for a funnel plot asymmetry test [26].

In the current recommendation for meta-analysis of diagnostic test accuracy from The Cochrane Collaboration, bivariate approach meta-analysis is preferred over the traditional univariate meta-analysis [17]. However, guidance for determining methodological approaches for meta-analysis with small numbers of studies is currently lacking. In this case, Doebler et al. and Takwoingi et al. encouraged the use of univariate approaches excluding pooling sensitivities and specificities [21, 27]. Eventually, both univariate and bivariate methods were conducted in the current study, and the diagnostic performance of ^18^F-FAMT against ^18^F-FDG was consistent under both approaches. The more conservative approach for HSROC estimation (Rücker-Schumacher’s method) also showed a similar tendency to the traditional HSROC parametrization (Rutter-Gatsonis’s method) [19].

Despite the limited number of studies included, results of our meta-analysis reflect the natural characteristics of both radiotracers that assess malignant lesions via different metabolic processes. The key feature of ^18^F-FDG is its superior capability to depict increased metabolic activity reflected by cell glucose consumption. The price of this high sensitivity is the detection accuracy that is prone to being obscured by normal physiological uptake, inflammation, and active benign tumors [2]. In a recent large-size meta-analysis, ^18^F-FDG PET failed to maintain its diagnostic accuracy for lung cancer in populations with endemic infectious lung disease [28]. ^18^F-FDG PET was also only moderately accurate for differentiating benign from malignant pleural effusions [29].

In another meta-analysis, whole-body ^18^F-FDG PET/CT remained superior to conventional imaging in the detection of distant malignancies, regardless of the primary tumor site and type [30]. However, the diagnostic accuracy of a PET radiotracer for lesions in the thorax and abdomen, where most primary lesions are located, is essential. It is well known that the role of ^18^F-FDG PET in oncology is often mitigated by many pitfalls, including background physiological uptake of major organs [31].

On the other hand, ^18^F-FAMT specific uptake depicted the actual malignant process. ^18^F-FAMT uptake reflects excessive transport of amino acids via LAT1, which is absent in normal cells and pathology other than malignancy [15]. However, the trade-off of ^18^F-FAMT’s high specificity is the relatively small absolute uptake in tumor cells, as a consequence of the nature of the LAT1 transporter. The influx of one amino acid substrate into tumor cells via LAT1 is mandatoryly coupled to the efflux of another amino acid substrate, resulting in ^18^F-FAMT’s relatively fast clearance from the tumor [14]. Nonetheless, the advantage of ^18^F-FAMT is the minimal background uptake in all organs except kidney and urinary tracts, allowing one to obtain high contrast images clearly depicting various types of malignancy including brain tumors [6, 13].

Meta-analyses evaluating the diagnostic performance of ^18^F-FDG PET in malignancy detection were mostly limited to a particular cancer type, or in comparison with conventional imaging (CT or MRI) or hybrid imaging (PET/CT or PET/MRI). Currently, only a few tumor-specific PET radiotracers are continuously investigated in a clinical setting for various type of cancers [32]. ^18^F-FET is probably the closest to ^18^F-FAMT in terms of chemical compound, radiochemistry, and clinical applicability. While ^18^F-FET has higher diagnostic accuracy than ^18^F-FDG, its effectiveness is limited for brain tumors [33]. l-[methyl-^11^C]-methionine (^11^C-MET), the most popular amino acid-based PET radiotracer to date, also has excellent diagnostic accuracy for glioma compared to ^18^F-FDG [34]. However, both ^18^F-FET and ^11^C-MET are also substrates for LAT2 transporters, which is also expressed in normal cells [14, 35]. The low kidney uptake PET tracer *anti*-1-amino-3-^18^F-fluorocyclobutane-1-carboxylic acid (^18^F-FACBC) has recently been meta-analyzed for its accuracy in prostate cancer recurrence detection. However, the specificity of ^18^F-FACBC is lower than ^11^C-choline PET and even T2-weighted MRI [36]. Therefore, ^18^F-FAMT probably the most versatile oncologic PET radiotracer currently available.

However, there a few limitations in this study and also in ^18^F-FAMT itself. First, all studies were from a single institution, which was potentially affected by publication bias despite the authors of each study belonging to various departments and evaluating different types of tumors. Even though studies by Watanabe et al. and Tian et al. focused on musculoskeletal tumors, they were separated by more than a decade, eliminating the possibility of overlapping patients [12, 23]. A study of various tumors by Inoue et al., however, included two patients with chondrosarcoma and schwannoma that might also be involved in the Watanabe et al. study, since these studies were from the same period [5, 12]. Unfortunately, this is difficult to confirm. Second, not all types of malignancies were evaluated; in particular, lymphoma, melanoma, pancreas and thyroid cancer, which are tumor types for which ^18^F-FDG PET is recommended to improve diagnostic accuracy [3]. Tumors in the pelvic area and abdomen were also poorly represented in this study.

Another drawback of the current ^18^F-FAMT studies is the absence of dynamic PET data. Currently ^18^F-FAMT PET scan is performed at 40–60 min post injection. However, phases as early as 5–15 min post injection might show higher tumor detection accuracy for any amino acid PET tracer considering the two-way-directional characteristic of amino acid uptake by their transporters [37]. A dynamic ^18^F-FAMT PET study in an animal tumor model showed that tumor-to-muscle uptake ratio is highest at 20 min and remains high at 60 min [38]. However, clinical dynamic PET studies are necessary to obtain optimal scan times.

Our current findings emphasize the need for prospective multicenter studies to overcome limitations of the single center report. This can only be achieved when the ^18^F-FAMT synthesis method is optimized and becomes widely used. The current ^18^F-FAMT radiofluorination method yields a low radioactivity that is only enough for PET scans for a mere three to four patients in each radiosynthesis [39]. Recently, a modified method of ^18^F-FAMT synthesis allows production to achieve high radioactivity for routine use [40]. However, a more practical approach is warranted. The twenty years of anticipation might soon be realized with the recent rapid development of fluorination methods. Of particular interest are the so-called late-stage fluorination methods which allow optimized synthesis of previously inaccessible PET radiotracers [41]. These novel radiofluorination approaches which make possible large-scale synthesis allow reconsideration of promising but underutilized radiotracers, like ^18^F-FAMT. Hence, revisiting the diagnostic performance of ^18^F-FAMT is a major step in the quest for an ideal general oncology PET tracer. Once these impediments are resolved, which we foresee shortly, the future may bring increased clinical impact of ^18^F-FAMT in oncology.

## Conclusion

^18^F-FAMT has diagnostic performance equal to or perhaps even better than ^18^F-FDG for malignancy detection in several cancer types. Future development in ^18^F-FAMT radiosynthesis might allow this tracer to be evaluated in other tumor types.

## Additional file

Additional file 1: Table S1. QUADAS tool assessment results (*n* = 9). **Table S2.** Diagnostic test results (DOCX 26 kb)

## Abbreviations

^11^C-MET: l-[methyl-^11^C] methionine
^18^F-FACBC: *anti*-1-amino-3-^18^F-fluorocyclobutane-1-carboxylic acid
^18^F-FAMT: l-3-^18^F-α-methyl tyrosine
^18^F-FDG: 2-deoxy-2-[^18^F]fluoro-d-glucose
^18^F-FET: *O*-(2-[^18^F]fluoroethyl)-l-tyrosine
DOR: Diagnostic odds ratio
LAT(1–4): L-type amino acid transporter (1–4)
PET: Positron emission tomography
QUADAS: Quality Assessment of Diagnostic Accuracy Studies
SPECT: Single-photon emission computed tomography
SROC: Summary receiver operating characteristics
SUV_max_: Maximum standardized uptake value

## References

[1] Farwell MD, Pryma DA, Mankoff DA (2014). PET/CT imaging in cancer: current applications and future directions. Cancer.

[2] Gillies RJ, Robey I, Gatenby RA (2008). Causes and consequences of increased glucose metabolism of cancers. J Nucl Med.

[3] Fletcher JW, Djulbegovic B, Soares HP, Siegel BA, Lowe VJ, Lyman GH (2008). Recommendations on the use of F-18-FDG PET in oncology. J Nucl Med.

[4] Huang C, McConathy J (2013). Radiolabeled amino acids for oncologic imaging. J Nucl Med.

[5] Inoue T, Koyama K, Oriuchi N, Alyafei S, Yuan Z, Suzuki H (2001). Detection of malignant tumors: whole-body PET with fluorine 18 alpha-methyl tyrosine versus FDG - preliminary study. Radiology.

[6] Inoue T, Shibasaki T, Oriuchi N, Aoyagi K, Tomiyoshi K, Amano S (1999). F-18 alpha-methyl tyrosine PET studies in patients with brain tumors. J Nucl Med.

[7] Kaira K, Oriuchi N, Otani Y, Yanagitani N, Sunaga N, Hisada T (2007). Diagnostic usefulness of fluorine-18-alpha-methyltyrosine positron emission tomography in combination with F-18-fluorodeoxyglucose in sarcoidosis patients. Chest.

[8] Kaira K, Oriuchi N, Shimizu K, Tominaga H, Yanagitani N, Sunaga N (2009). F-18-FMT uptake seen within primary cancer on PET helps predict outcome of non-small cell lung cancer. J Nucl Med.

[9] Miyakubo M, Oriuchi N, Tsushima Y, Higuchi T, Koyama K, Arai K (2007). Diagnosis of maxillofacial tumor with L-3-[F-18]-fluoro-alpha-methyltyrosine (FMT) PET: a comparative study with FDG-PET. Ann Nucl Med.

[10] Sohda M, Sakai M, Honjyo H, Hara K, Ozawa D, Suzuki S (2014). Use of pre-treatment F-18-FAMT PET to predict patient survival in Squamous cell carcinoma of the esophagus treated by curative surgery. Anticancer Res.

[11] Suzuki R, Watanabe H, Yanagawa T, Sato J, Shinozaki T, Suzuki H (2005). PET evaluation of fatty tumors in the extremity: possibility of using the standardized uptake value (SUV) to differentiate benign tumors from liposarcoma. Ann Nucl Med.

[12] Watanabe H, Inoue T, Shinozaki T, Yanagawa T, Ahmed AR, Tomiyoshi K (2000). PET imaging of musculoskeletal tumours with fluorine-18 alpha-methyltyrosine: comparison with fluorine-18 fluorodeoxyglucose PET. Eur J Nucl Med.

[13] Sato N, Inoue T, Tomiyoshi K, Aoki J, Oriuchi N, Takahashi A (2003). Gliomatosis cerebri evaluated by F-18 alpha-methyl tyrosine positron-emission tomography. Neuroradiology.

[14] Wiriyasermkul P, Nagamori S, Tominaga H, Oriuchi N, Kaira K, Nakao H (2012). Transport of 3-Fluoro-L-alpha-methyl-tyrosine by tumor-Upregulated L-type amino acid transporter 1: a cause of the tumor uptake in PET. J Nucl Med.

[15] Wei L, Tominaga H, Ohgaki R, Wiriyasermkul P, Hagiwara K, Okuda S (2016). Specific transport of 3-fluoro-l-α-methyl-tyrosine by LAT1 explains its specificity to malignant tumors in imaging. Cancer Sci.

[16] Reitsma JB, Moons KGM, Bossuyt PMM, Linnet K (2012). Systematic reviews of studies quantifying the accuracy of diagnostic tests and markers. Clin Chem.

[17] Macaskill P, Gatsonis C, Deeks J, Harbord R, Takwoingi Y. Chapter 10: Analysing and Presenting Results. In: Cochrane Handbook for Systematic Reviews of Diagnostic Test Accuracy Version 1. Deeks J, Bossuyt P, Gatsonis C, editors. The Cochrane Collaboration. 2010. http://methods.cochrane.org/sdt/handbook-dta-reviews. Accessed 15 Dec 2015.

[18] Whiting PF, Rutjes AW, Westwood ME, Mallett S, Deeks JJ, Reitsma JB (2011). QUADAS-2: a revised tool for the quality assessment of diagnostic accuracy studies. Ann Intern Med.

[19] Rücker G, Schumacher M (2010). Summary ROC curve based on a weighted Youden index for selecting an optimal cutpoint in meta-analysis of diagnostic accuracy. Stat Med.

[20] Glas AS, Lijmer JG, Prins MH, Bonsel GJ, Bossuyt PMM (2003). The diagnostic odds ratio: a single indicator of test performance. J Clin Epidemiol.

[21] Doebler P (2015). Mada: meta-analysis of diagnostic accuracy. R package version 0.5.7.

[22] R-Core-Team (2015). R: a language and environment for statistical computing.

[23] Tian M, Zhang H, Endo K. Comparison of cell proliferation, protein, and glucose metabolism in musculoskeletal tumors in a PET study. J Biomed Biotechnol. 2011; 10.1155/2011/807929. 10.1155/2011/807929 PMC3119418 21738405

[24] Kaira K, Oriuchi N, Shimizu K, Ishikita T, Higuchi T, Imai H (2009). Evaluation of thoracic tumors with F-18-FMT and F-18-FDG PET-CT: a clinicopathological study. Int J Cancer.

[25] Lijmer JG, Bossuyt PMM, Heisterkamp SH (2002). Exploring sources of heterogeneity in systematic reviews of diagnostic tests. Stat Med.

[26] Bürkner P-C, Doebler P (2014). Testing for publication bias in diagnostic meta-analysis: a simulation study. Stat Med.

[27] Takwoingi Y, Guo B, Riley RD, Deeks JJ. Performance of methods for meta-analysis of diagnostic test accuracy with few studies or sparse data. Stat Methods Med Res. 2015; 10.1177/0962280215592269. 10.1177/0962280215592269 PMC5564999 26116616

[28] Deppen SA, Blume JD, Kensinger CD, Morgan AM, Aldrich MC, Massion PP (2014). Accuracy of FDG-PET to diagnose lung cancer in areas with infectious lung disease a meta-analysis. JAMA.

[29] Porcel JM, Hernandez P, Martinez-Alonso M, Bielsa S, Salud A (2015). Accuracy of Fluorodeoxyglucose-PET imaging for differentiating benign from malignant pleural effusions a meta-analysis. Chest.

[30] Xu G, Zhao L, He Z (2012). Performance of whole-body PET/CT for the detection of distant malignancies in various cancers: a systematic review and meta-analysis. J Nucl Med.

[31] Corrigan AJG, Schleyer PJ, Cook GJ (2015). Pitfalls and artifacts in the use of PET/CT in oncology imaging. Semin Nucl Med.

[32] Rice SL, Roney CA, Daumar P, Lewis JS (2011). The next generation of positron emission tomography radiopharmaceuticals in oncology. Semin Nucl Med.

[33] Dunet V, Rossier C, Buck A, Stupp R, Prior JO (2012). Performance of F-18-Fluoro-ethyl-tyrosine (F-18-FET) PET for the differential diagnosis of primary brain tumor: a systematic review and Metaanalysis. J Nucl Med.

[34] Zhao C, Zhang Y, Wang J (2014). A meta-analysis on the diagnostic performance of ^18^F-FDG and ^11^C-methionine PET for differentiating brain tumors. AJNR Am J Neuroradiol.

[35] Habermeier A, Graf J, Sandhofer BF, Boissel JP, Roesch F, Closs EI (2015). System L amino acid transporter LAT1 accumulates O-(2-fluoroethyl)-L-tyrosine (FET). Amino Acids.

[36] Ren J, Yuan L, Wen G, Yang J (2016). The value of anti-1-amino-3-^18^F-fluorocyclobutane-1-carboxylic acid PET/CT in the diagnosis of recurrent prostate carcinoma: a meta-analysis. Acta Radiol.

[37] Kameyama M, Umeda-Kameyama Y (2016). Strategy based on kinetics of O-(2-[^18^F] fluoroethyl)-L-tyrosine ([^18^F] FET). Eur J Nucl Med Mol Imaging.

[38] Yamaguchi A, Hanaoka H, Fujisawa Y, Zhao S, Suzue K, Morita A (2015). Differentiation of malignant tumours from granuloma by using dynamic [^18^F]-fluoro-L-α-methyltyrosine positron emission tomography. EJNMMI Res.

[39] Tomiyoshi K, Amed K, Muhammad S, Higuchi T, Inoue T, Endo K (1997). Synthesis of isomers of ^18^F-labelled amino acid radiopharmaceutical: position 2- and 3-L-^18^F-α-methyltyrosine using a separation and purification system. Nucl Med Commun.

[40] Meleán JC, Humpert S, Ermert J, Coenen HH (2015). Stereoselective radiosynthesis of L- and D-3-[F-18]fluoro-alpha-methyltyrosine. J Fluor Chem.

[41] Preshlock S, Tredwell M, Gouverneur V (2016). ^18^F-labeling of Arenes and Heteroarenes for applications in positron emission tomography. Chem Rev.

