# Supplementary material for: The diagnostic performance of 18F-FAMT PET and 18F-FDG PET for malignancy detection: a meta-analysis
Source: BMC Med Imaging. 2017 Dec 28;17:66. doi: 10.1186/s12880-017-0237-1 (PMC5745915; doi:10.1186/s12880-017-0237-1)
Supplement: Additional file 1: Table S1. — QUADAS tool assessment results (n = 9). Table S2. Diagnostic test results (DOCX 26 kb) [file 12880_2017_237_MOESM1_ESM.docx]

**Additional files**

**Table S1.** QUADAS tool assessment results (*n* = 9)

**Table S2.** Diagnostic test results

| **Studies**  **(First Author, Year)** |  | **Based on Visual Assessment** | | | | | | | | |  | **Based on Diagnostic Cut-off Values** | | | | | | | | | | |
| --- | --- | --- | --- | --- | --- | --- | --- | --- | --- | --- | --- | --- | --- | --- | --- | --- | --- | --- | --- | --- | --- | --- |
|  |  | **^18^F-FAMT** | | | |  | **^18^F-FDG** | | | |  | **^18^F-FAMT** | | | | |  | **^18^F-FDG** | | | | |
|  |  | **TP** | **FP** | **FN** | **TN** |  | **TP** | **FP** | **FN** | **TN** |  | **TP** | **FP** | **FN** | **TN** | **cut off** |  | **TP** | **FP** | **FN** | **TN** | **cut off** |
| Watanabe (2000)^12^ |  | 22 | 39 | 0 | 14 |  | 22 | 45 | 0 | 8 |  | 16 | 8 | 6 | 45 | **1.20** |  | 16 | 19 | 6 | 34 | **1.90** |
| Inoue (2001)^5^ |  | 26 | 4 | 9 | 18 |  | 32 | 19 | 3 | 3 |  | - | - | - | - | **-** |  | - | - | - | - | **-** |
| Suzuki (2005)^11^ |  | 6 | 1 | 3 | 11 |  | 8 | 3 | 1 | 9 |  | 6 | 1 | 3 | 11 | **1** |  | 8 | 3 | 1 | 9 | **0.81** |
| Miyakubo (2007)^9^ |  | 30 | 2 | 6 | 8 |  | 29 | 2 | 7 | 8 |  | 30 | 2 | 6 | 8 | **1.45** |  | 29 | 2 | 7 | 8 | **4.72** |
| Kaira (2009)^24^ |  | 31 | 0 | 6 | 6 |  | 33 | 5 | 4 | 1 |  | - | - | - | - | **-** |  | - | - | - | - | **-** |
| Tian (2011)^23^ |  | 11 | 22 | 2 | 1 |  | 13 | 23 | 0 | 0 |  | 8 | 4 | 5 | 19 | **1.26** |  | 10 | 7 | 3 | 16 | **2.77** |
